# Phenotypic Characterization of *Sulfolobus islandicus* Strains Lacking the B-Family DNA Polymerases PolB2 and PolB3 Individually and in Combination

**DOI:** 10.3389/fmicb.2021.666974

**Published:** 2021-04-22

**Authors:** Peter B. Bohall, Stephen D. Bell

**Affiliations:** ^1^Department of Molecular and Cellular Biochemistry, Indiana University, Bloomington, IN, United States; ^2^Department of Biology, Indiana University, Bloomington, IN, United States

**Keywords:** Sulfolobus, archaea, DNA replication, DNA damage, polymerase

## Abstract

Across the three domains of life, B-family DNA polymerases play a variety of roles in both DNA repair and DNA replication processes. We examine the phenotypic consequences of loss of the putative repair polymerases PolB2 and/or PolB3 in the crenarchaeon *Sulfolobus islandicus*. We detect a modest growth advantage when cells lacking the polymerase are grown in unperturbed conditions. Further, we observe a striking insensitivity of the mutant lines to acute treatment with the oxidizing agent, hydrogen peroxide. In addition, cells lacking PolB3 show enhanced sensitivity to the DNA damaging agent 4-NQO. Our data therefore suggest that these non-essential DNA polymerases may influence DNA repair pathway choice in these hyperthermophilic aerobes.

## Introduction

DNA polymerases perform the essential function of genome replication but show striking diversity across phyletic divides. Even within a domain of life there is considerable variation in the representation of different DNA polymerase family members. For example, most archaea possess a signature D-family DNA polymerase, PolD, that has recently been shown to possess a double-psi barrel catalytic fold reminiscent of multi-subunit RNA polymerases. The D-family enzymes are heterodimers and features of the interaction interface between subunits are conserved in eukaryotic multi-subunit DNA polymerases (Tahirov et al., 2009; Raia et al., 2019a, b; Greci and Bell, 2020). Interestingly, the PolD-containing archaea also encode a B-family DNA polymerase, PolB. The available evidence suggests that PolD is responsible for the majority of genome replication with PolB playing a non-essential role, most likely in DNA repair processes (Cubonova et al., 2013; Sarmiento et al., 2013).

The Crenarchaea, which harbor the model organisms of the *Sulfolobales* lack a D-family polymerase. With a single exception, species within the *Sulfolobales* typically encode three B-family polymerases as well as a Y-family DNA polymerase that is dedicated to DNA repair (Miyabayashi et al., 2020). Recent work has revealed that of the three PolB enzymes, only PolB1 is essential for viability, while cells lacking PolB2 or PolB3 are able to grow (Martinez-Alvarez et al., 2017). Indeed, a recent characterization of *Sulfodiicoccus acidiphilus* has revealed that this species naturally lacks PolB3 (Sakai and Kurosawa, 2019). It has long been known that the gene for PolB2 is transcriptionally induced in response to UV-induced stress on cells (Frols et al., 2007; Gotz et al., 2007). However, little is known about the physiological role of PolB3. Notably, PolB3 is the closest sequence relative to the PolB putative DNA repair enzyme in those archaea that possess the PolD replicative DNA polymerase (Makarova et al., 2014). Thus, it seems likely that both PolB2 and PolB3 play roles in the DNA damage response. Recent work has provided evidence to support this proposal, with strains lacking PolB2 or PolB3 demonstrating sensitivity to DNA damaging agents (Feng et al., 2020; Miyabayashi et al., 2020). In the following, we address the roles of PolB2 and/or PolB3 during normal, unperturbed growth and under conditions of acute and chronic treatment with an array of DNA damaging agents.

## Materials and Methods

### Archaeal Strains

The *dpo2* and *dpo3* knock-out strains used in these experiments were provided by Prof. Xu Peng, Copenhagen (Martinez-Alvarez et al., 2017). The *dpo2/3* knock-out was constructed by a modification of the *S. islandicus* type I CRISPR-Cas3 system as previously described (Li et al., 2016; see Table 1).

**TABLE 1:** Strains used in this study.

**Strains**	**Genotype**	**Source**
*S. islandicus* LAL14/1-CD	*pyrEF* disruption mutant	Jaubert et al., 2013
*S. islandicus* Dpo2KO	*S. islandicus* LAL14/1 E233S lacking the *dpo2* gene, deletion from position 635,294–637,207 in the *S. islandicus* LAL14/1 genome	Martinez-Alvarez et al., 2017
*S. islandicus* Dpo3KO	*S. islandicus* LAL14/1 E233S lacking the *dpo3* gene, deletion from position 1,742,652–1,744,934 in the *S. islandicus* LAL14/1 genome	Martinez-Alvarez et al., 2017
*S. islandicus* Dpo2/3KO	*S. islandicus* LAL14/1 E233S lacking both the *dpo2* and *dpo3* genes	This work

**TABLE 2:** Plasmids used in this study.

**Plasmids**	**Features**	**Source**
pGE1	Genome-editing plasmid containing tandem CRISPR repeats for constructing an artificial mini-CRISPR loci and *Sac* pyrE and pyrF genes	Li et al., 2016
pGE1s	pGE1 modified to contain the *Sto* HMG-CoA gene for Simvastatin selection	This work
pGE1s-Dpo2KO	pGE1s including Donor DNA from regions in the *Sis* genome flanking *dpo2* and a mini-CRISPR locus with a spacer selected from an internal region of Dpo2	This work
pGE1s-Dpo3KO	pGE1s including Donor DNA from regions in the *Sis* genome flanking *dpo3* and a mini-CRISPR locus with a spacer selected from an internal region of Dpo3	This work

To improve the selection of transformants we modified the pGE1 plasmid (Martinez-Alvarez et al., 2017) to include the *Sulfolobus tokodaii* gene for HMG-CoA reductase, the over-expression of which confers simvastatin resistance in Sulfolobus species (Zheng et al., 2012), by amplification of the cassette from pSSR (Zheng et al., 2012) using oligonucleotides ForSimR and RevSimR (see Table 3), and insertion into the SmaI site of pGE1 (we note that we have had persistent problems with high background when relying on uracil prototrophy conferred by the original pGE1 vector). Initial transformants were selected using 18 μM simvastatin and subsequently grown without the drug and in the presence of 20 μg/ml 5-Fluoroorotic Acid (5-FOA) to counter select. All strains, plasmids and DNA oligonucleotides used are listed in Tables 1–3. Competent cells were made by washing harvested mid-log phase cells 3 times with 20 mM sucrose at room temperature. Six hundred nanogram of vector were introduced by means of electroporation via a single pulse using a BioRad GenePulserXcell and BioRad Shock Pod with parameters set to 1.2 kV, 25 μF, and 600 Ω.

**TABLE 3:** Oligonucleotides used in this study.

**Oligonucleotides Used**	**Sequences (5′–3′)**
Dpo2 SphI-Left F	CATGCATGCCCTCATAAAGGTATTGGAGA
Dpo2 XhoI-Right R	CCGCTCGAGAACAAACCTCCATCATCACTT
Dpo2 SpF	AAGAAGCAATTTCAAGGAGAAAAGTATCAGAACAACGTTCCCA
Dpo2 SpR	AGCTGGGAACGTTCTGATACTTTTCTCCTTGAAATTGCTT
Dpo3 SphI-Left F	CATGCATGCTCCGAGAGTATCTTTATCCCT
Dpo3 XhoI-Left R	CCGCTCGAGTTAGACAGGATTGAGACTGC
Dpo3 SpF	AAGCTAATTTACATTTGGAGCATTGATGATGAAGGTAACAGTT
Dpo3 SpR	AGCAACTGTTACCTTCATCATCAATGCTCCAAATGTAAATTAG
LAL-Check Dpo2 F	GCGGAAGCGGAGGACTATT
LAL-Check Dpo2 R	CGTAAACTGGGGCTGAAATGG
LAL-Check Dpo3 F	CTAGTGGCCGATGATACGCT
LAL-Check Dpo3 R	TGAGAAAGTTCAAGTGCGAGA
ForSimR	CTAATTGCGGCCGCCCCCTCACTATAACTAGCTAGTTTAAG
RevSimR	TTATATCCCGGGAACTTTTAAACTTTGGCCCCTC

*Restriction enzyme sites are underlined.*

### Growth Conditions

WT and mutant strains were grown in Tryptone-Sucrose-Vitamin-Yeast Extract (TSVY) medium [0.1% tryptone (w/v), 0.2% sucrose (w/v), 1% vitamin solution (v/v), 0.05% Yeast Extract (w/v), pH 3.5], or TSVYu (+20 μg/ml uracil) at 78°C. Transformations were grown on Sucrose-Casamino Acid-Vitamin-Yeast Extract (SCVy) plates with 18 μM simvastatin [0.2% sucrose (w/v), 0.2% Casamino Acids (w/v), 0.004% Yeast Extract (w/v), pH 3.5] at 78°C. All plating was carried out on equivalent medium plates containing 1% Gelrite. For growth curve analysis, 50–100 ml cultures were started at *A*_600_ = 0.03 in media pre-warmed to 78°C. The growth was monitored by tracking *A*_600_ values with an Eppendorf BioPhotometer at 6 h intervals.

### Flow Cytometry

One hundred microliter samples of cell culture were collected at 6 h intervals during growth. The cells were then fixed in 900 μL ice-cold 80% ethanol and stored at 4°C for up to 1 month. 500 μL of fixed cells were subsequently centrifuged for 5 min at 15,000 rpm and washed in 500 μL sterile 10 mM Tris-HCl (pH 7.4), 10 mM MgCl_2_, followed by a second wash in 250 μL of the same buffer. Cells were centrifuged again and resuspended in 1 mL of the same buffer containing 2.5 μM Sytox Green (Invitrogen) and 5 μg/mL RNase A. Analysis of samples was performed on a BD LSR II flow cytometer using FACSDiva software and a laser excitation of 488-nm. A data set of at least 50,000 events was collected for each sample.

### Genomic DNA Preparation and MFA-Seq

Five to twenty milliliter cell culture were harvested and washed 2 times with TEN buffer [50 mM Tris-HCl (pH8.0), 50 mM EDTA, 100 mM NaCl] and resuspended in 1/10 the original volume. 20% SDS solution was added to a final concentration of 0.1% along with RNase A (0.02 mg/ml) and incubated at 37°C for 30 min, followed by the addition of proteinase K (0.02 mg/ml) and a 1 h incubation at 37°C. The lysate was extracted in 1 volume phenol:chloroform:isoamyl alcohol, 25:24:1 (pH 7.8), 2 times, followed by 1 volume of chloroform alone. Genomic DNA was then precipitated in 66% ethanol and analyzed by 0.8% agarose gel electrophoresis. Marker Frequency Analysis (MFA-Seq) was performed by Illumina sequencing of DNA from exponentially growing or stationary phase cells. Between 17 and 22 million reads per sample were mapped to the *S. islandicus* LAL14/1 reference genome using the program Geneious Prime version 2020.2.2. BAM files generated by Geneious were analyzed using SeqMonk^1^. Read counts were generated in 2 kb probe windows and filtered to exclude repetitive elements. Read counts per probe were normalized by reference to stationary phase (non-replicating) DNA to account for variation in sequencing efficiency across the genome. SNPs were identified using Geneious.

### Drug Preparation

Each drug that was used was prepared from powder or a stable concentrated stock immediately before its addition to the media. Hydrogen Peroxide solution was prepared by diluting 35% H_2_O_2_ to a concentration of 127 mM in water. Hydroxyurea was prepared from powder to a working concentration of 1 M in water. 4-Nitroquinoline 1-oxide (4-NQO) was first prepared as a concentrated solution from powder in DMSO, it was then diluted to a working concertation of 130 μM in 1% DMSO. Cisplatin was prepared from powder to a working concentration of 0.5 mg/ml in 0.9% NaCl solution.

### Exposure to DNA Damaging Agents and Spot Assays

For acute exposure to Hydrogen Peroxide, Hydroxyurea, and 4-NQO, strains were first grown to *A*_600_ = 0.3–0.4, all strains were then normalized to *A*_600_ = 0.3 by the addition of pre-warmed media and split into 10 ml cultures before the indicated amount of drug was added. The cells were returned to 78°C and grown with shaking (110 rpm) for 7 h before samples were collected for plating or inoculating new cultures. Spot plating was accomplished by diluting each culture to *A*_600_ = 0.1 in pre-warmed TSVY, followed by serial diluting twofold or 10-fold as indicated. Each dilution was kept at 78°C with 5 μl spotted onto TSVYu plates. Plates were grown at 78°C for 7–8 days. For the 4-NQO recovery assay, cells were washed with fresh TSVYu after acute drug exposure as described above, then used to inoculate cultures to A_600_ = 0.03. The cultures were subsequently grown at 78°C with growth tracked by *A*_600_ every 6 h until cell death was observed. UV exposure was accomplished by growing each strain to *A*_600_ = 0.1, after which 3 ml were used to cover the bottom of a 100 mm × 15 mm polystyrene petri dish at room temperature. Cells were exposed to 200 and 250 J/m^2^ UV (additional UV doses were also checked; data not shown) using a SpectroLinker XL-1000 UV Crosslinker (Spectronics Corporation). Plating was carried out as described above in a dark room to prevent photo-reactivation. For chronic exposure to Hydrogen Peroxide, Hydroxyurea, 4-NQO, and Cisplatin, strains were grown to *A*_600_ = 0.3–0.4, then diluted to *A*_600_ = 0.1 in pre-warmed TSVY. Samples were diluted and spotted as previously described onto TSVYu plates containing the indicated concentration of drug. Plates were grown at 78°C for 7–8 days.

## Results

### The Impact of Loss of *dpo2* and/or *dpo3* on Unperturbed Growth

**FIGURE 1:**
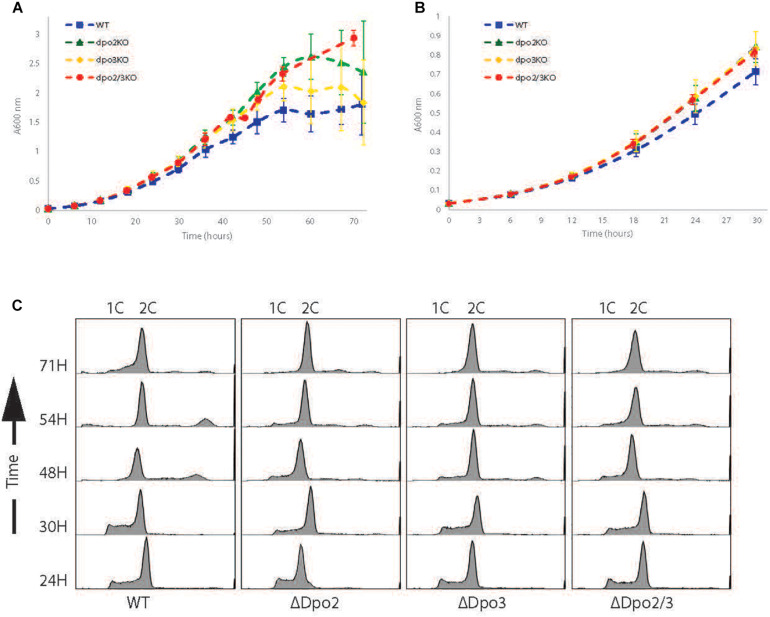
Growth profiles of strains. **(A)** Growth curve of WT, Dpo2KO, Dpo3KO, and Dpo2/3KO lines over 72 h. Samples were collected for analysis every 6 h. Growth curves for each line were repeated at least 3 times. **(B)** The first 30 h growth for each line. **(C)** Flow cytometry profiles of each line. Samples collected simultaneously with growth curve samples.

**FIGURE 2:**
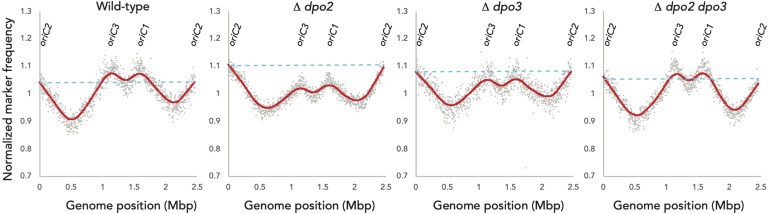
Marker-Frequency Analysis. Marker-frequency analysis of WT, Δ*dpo2*,Δ*dpo3, and*Δ*dpo2/3* lines. DNA was collected from each culture during exponential phase growth and subjected to Illumina next-generation sequencing. Read counts were generated in 2 kb windows and normalized to stationary phase DNA. Raw values for individual counts are displayed in gray and a LOWESS smoothing is shown in red. The dotted blue line indicates the amplitude of the *oriC2* peak.

We focused our efforts on the *Sulfolobus islandicus* strain LAL14/1. Previous work by Peng and colleagues had revealed PolB1 to be essential for viability in this strain (Martinez-Alvarez et al., 2017). However, strains lacking the *dpo2* or *dpo3* genes, that encode PolB2 or PolB3, respectively, were able to survive. We obtained these mutant strains and, using the endogenous CRISPR system (Li et al., 2016), generated a further strain that lacked both PolB2 and PolB3. We first measured the growth parameters of the wild-type and three mutant strains under standard, non-perturbed conditions in rich medium (Figure 1A). During exponential growth both single mutants showed slightly faster growth rates than wild-type. More specifically, the doubling time of both mutants was 6.3 h, compared to 6.7 h for wild-type (Figures 1A,B). Furthermore, the single mutants entered stationary phase at higher cell densities than did the wild-type cultures and showed considerable variation in cell numbers at analogous times between replicates in stationary phase. The double mutant had the same accelerated growth rate during exponential growth as the single mutants (doubling time of 6.3 h) and entered stationary phase at a higher cell density than observed with the wild-type. We analyzed the cell cycle distribution of the wild-type and mutant lines using flow cytometry but could not observe any reproducible differences in the population of G1, S, and G2 phase cells between wild-type and mutants (Figure 1C). We next analyzed DNA replication profiles by Marker Frequency Analysis as measured by Illumina Sequencing (MFA-Seq). MFA-Seq profiles indicated that all four cell lines initiated DNA replication from the three DNA replication origins conserved in *Sulfolobus* species (Figures 2A–D). In agreement with our previous analyses on other *Sulfolobus* species and strains (Duggin et al., 2008; Samson et al., 2013), in wild-type cells the amplitude of the peak corresponding to *oriC2* is lower than that for *oriC1* and *oriC3* (dotted blue line in Figure 2). We have previously demonstrated that this is due to slightly later firing of *oriC2* in S-phase (Duggin et al., 2008). Interestingly, examination of the data reveal that the peaks corresponding to initiation at *oriC2* are higher than those seen for *oriC1* and *oriC3* in cells lacking either PolB2 or PolB3. Furthermore, the double mutant has a profile closer to that of wild-type, with all three origins giving peaks of similar amplitude. We speculate that while the gross cell cycle parameters of the mutant cell lines are essentially unaltered, perhaps the accelerated firing of *oriC2* is indicative of some subtle mis-regulation linked to the accelerated growth rate of these strains. However, this remains pure speculation at this time. The MFA-Seq data also allowed us to determine if any unanticipated mutations had arisen in any of the strains. Comparison of our laboratory wild-type strain of *S. islandicus* LAL14/1, obtained from Prof. Xu Peng, Copenhagen, revealed 23 SNPs relative to the reference genome sequence deposited at NCBI (Jaubert et al., 2013; Table 4). A total of 13 further SNPs were detected in the genome of the mutant strains, not including those associated with the targeted mutations (Table 5). Eight of those resulted in synonymous codon changes. Four of the remaining five were in multi-copy transposable element-encoded genes (and could be artifacts due to alignment errors to these repetitive elements) and the final mutation was a conservative mutation in the *dpo2* knock-out strain resulting in a M-L substitution in the SiL_0710 open-reading frame. Notably, this same substitution is seen naturally in a second copy of this gene (SiL_0897). Accordingly, we can be confident that the phenotypes we observe arise as a consequence of deletion of the targeted DNA polymerase genes.

**TABLE 4:** SNPs identified in the wild-type strain (compared to the *S. islandicus* LAL14/1 reference genome: NCBI NC_021058.1).

**Name**	**Minimum**	**Amino acid change**	**Change**	**Codon change**	**Locus_Tag**	**Polymorphism type**	**Protein effect**	**Variant frequency**	**Variant *P*-value (approximate)**	**Protein_Id**
C	428,853		A → C	CTA → CTC	SIL_RS02310	SNP (transversion)	None	100.00%	0	WP_012713028.1
T	585,179		A → T			SNP (transversion)		100.00%	0	
A	623,184	W → R	T → A	TGG → AGG	SIL_RS03215	SNP (transversion)	Substitution	100.00%	4.00E–09	WP_048050180.1
G	786,599	W → G	T → G	TGG → GGG	SIL_RS04120	SNP (transversion)	Substitution	99.70%	0	WP_015580995.1
C	854,359		T → C	ACA → ACG	SIL_RS04480	SNP (transition)	None	92.90%	8.80E–44	WP_015581066.1
AGACATTA	1,012,613	PMVG → PDIS	TATGGTGG → AGACATTA	CCT,ATG,GTG,GGC → CCA,GAC,ATT,AGC	SIL_RS05320	Substitution	Substitution	96.7% → 100.0%	6.30E–08	
C	1,012,649		A → C	GTA → GTC	SIL_RS05320	SNP (transversion)	None	100.00%	6.30E–113	
A	1,012,652	F → L	C → A	TTC → TTA	SIL_RS05320	SNP (transversion)	Substitution	100.00%	1.60E–109	
A	1,012,658	F → L	C → A	TTC → TTA	SIL_RS05320	SNP (transversion)	Substitution	98.60%	7.10E–216	
C	1,012,662	S → P	T → C	TCA → CCA	SIL_RS05320	SNP (transition)	Substitution	99.20%	0	
G	1,301,868	F → S	A → G	TTT → TCT	SIL_RS06995	SNP (transition)	Substitution	100.00%	0	WP_012711510.1
T	1,301,873	N → K	A → T	AAT → AAA	SIL_RS06995	SNP (transversion)	Substitution	100.00%	0	WP_012711510.1
	1,301,880		Deletion		SIL_RS06995	Deletion	Frame Shift	90.50%	1.70E–53	WP_012711510.1
GC	1,301,885	Y → C	AT → GC	TAT → TGC	SIL_RS06995	Substitution	Substitution	100.00%	1.00E–16	WP_012711510.1
TTA	1,302,407		CTG → TTA		SIL_RS06995	Substitution	Truncation	100.00%	1.60E–92	WP_012711510.1
GCTGC	1,302,412	LSY → LQH	ATGAG → GCTGC	CTC,TCA,TAT → CTG,CAG,CAT	SIL_RS06995	Substitution	Substitution	98.9% → 100.0%	2.50E–201	WP_012711510.1
G	1,574,824	M → V	A → G	ATG → GTG	SIL_RS08590	SNP (transition)	Substitution	100.00%	0	WP_015581345.1
C	1,866,459	V → A	T → C	GTT → GCT	SIL_RS10225	SNP (transition)	Substitution	100.00%	0	WP_014514148.1
C	2,171,368	N → D	T → C	AAT → GAT	SIL_RS11740	SNP (transition)	Substitution	100.00%	0	WP_015581604.1
G	2,360,056		A → G		SIL_RS12715	SNP (transition)	Extension	100.00%	0	
A	2,369,118	S → F	G → A	TCT → TTT	SIL_RS12760	SNP (transition)	Substitution	99.70%	0	WP_012714645.1
A	2,388,397	Q → K	C → A	CAG → AAG	SIL_RS12855	SNP (transversion)	Substitution	100.00%	2.50E–14	WP_015581719.1
A	2,388,411		C → A		SIL_RS12855	SNP (transversion)	Truncation	100.00%	6.30E–20	WP_015581719.1

**TABLE 5:** SNPs identified in the Δ*dpo* mutant strains that were not in the wild-type.

**SNP**	**Coordinate**	**Change**	**Codon change**	**Protein effect**	**Variant frequency**	**Variant *P*-value (approximate)**	**Present in strains**	**Protein_id**	**Protein name**
1	333,955	G → A	GGG → AGG	Substitution	100.00%	2.50E–07	dpo3 KO	WP_015581754.1	IS200/IS605 TnpB
2	364,496	G → T	CTC → CTA	None	100.00%	1.00E–07	dpo2 KO, double mutant	WP_015580753.1	Transposase
3	364,556	G → A	GAC → GAT	None	100.00%	3.20E–18	dpo2 KO, dpo3 KO, double mutant	WP_015580753.1	Transposase
4	364,560	T → C	AAT → AGT	Substitution	100.00%	1.60E–24	dpo2 KO, dpo3 KO, double mutant	WP_015580753.1	Transposase
5	364,560	T → C	GAA → GAG	None	100.00%	1.60E–24	dpo2 KO, dpo3 KO, double mutant	WP_014513344.1	IS200/IS605 Transposase
6	364,620	T → A	GCA → GCT	None	100.00%	1.00E–17	dpo2 KO, dpo3 KO	WP_014513344.1	IS200/IS605 Transposase
7	364,625	G → A	CTA → TTA	None	100.00%	1.00E–14	dpo2 KO, dpo3 KO	WP_014513344.1	IS200/IS605 Transposase
8	364,629	A → G	TAT → TAC	None	100.00%	2.50E–12	dpo2 KO, dpo3 KO	WP_014513344.1	IS200/IS605 Transposase
9	364,680	A → G	TTT → TTC	None	100.00%	2.50E–07	dpo2 KO	WP_014513344.1	IS200/IS605 Transposase
10	364,683	TT → CC	AAA → AGG	Substitution	100.00%	3.20E–11	dpo2 KO	WP_014513344.1	IS200/IS605 Transposase
11	677,631	A → T	ATG → TTG	Substitution	100.00%	1.00E–07	dpo2 KO	WP_048050189.1	DUF1286 domain containing protein
12	765,000	C → T	GGA → AGA	Substitution	100.00%	4.00E–39	Double mutant	WP_010923529.1	ISH3 transposase
13	1,915,739	T → C	CCA → CCG	None	99.70%	0	dpo2 KO, dpo3 KO, double mutant	WP_048050389.1	MFS Transporter

### Sensitivity to Acute DNA Damage

**FIGURE 3:**
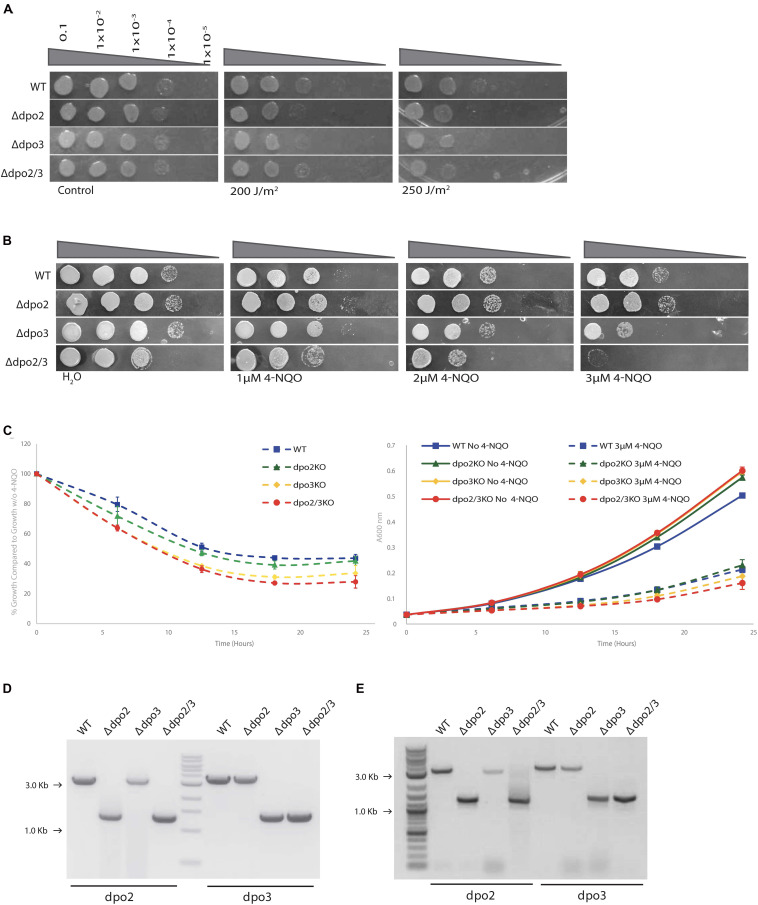
Growth of strains exposed to UV and acute 4-NQO. **(A)** Serial dilutions of each cell line plated onto TSVYu after exposure to UV (254 nm) at the indicated intensity. Growth conditions and spot plates were repeated at least 3 times. **(B)** Serial dilutions of each line plated onto TSVYu after growth for 7 h (about 1 doubling time) with 4-NQO at the indicated concentrations. Growth conditions and spot plates were repeated at least 3 times. **(C)** Growth analysis of cell lines grown in TSVYu media after growth for 7 h with 3 μM 4-NQO. Cultures were grown over a 24 h period starting at *A*_600_ = 0.03 with samples collected for analysis ever 6 h. In the left hand panel, *A*_600_ values were normalized against growth without 4-NQO. The right hand panel shows the original growth curves. **(D)** Agarose gel electrophoresis of PCR tests confirming that our freezer stocks possessed anticipated genotype, positions of PCR products indicating amplification of wild-type (WT) or deletion-containing (Δ) loci are indicated (M-DNA markers). **(E)** As D but with DNA isolated from cells derived from the plates shown **(B)**.

First, we tested the effect of exposure of cells to 0, 200, or 250 J/m^2^ of UV light of 254 nm (Figure 3A). A modest effect was observed at the highest UV dose, with the *dpo2/3* double mutant showing reduced survival in spot tests of serial dilutions of cells following exposure (to prevent photoreactivation these experiments and growth of plates were performed in the dark). We also tested the effect of acute exposure to 4-Nitroquinoline-1 oxide (4-NQO), which, like UV, induces lesions typically repaired by nucleotide excision repair pathways, by administering the drug at 0, 1, 2, or 3 μM) for 7 h (roughly one doubling time) prior to washing the cells and assaying survival by serial dilution and plating. Loss of PolB3 sensitized cells to 4-NQO while loss of PolB2 had no discernable effect. Interestingly, however, the double mutant was more sensitive to 2 and 3 μM 4-NQO than either mutant alone (Figure 3B). We additionally compared growth assays in liquid culture of cells either mock treated or transiently treated with 4-NQO. In agreement with the plating assays, we observed that loss of PolB2 had minimal effects, loss of PolB3 sensitized cells to 4-NQO and the double mutant strain was most sensitive of all (Figure 3C). This suggests that PolB3 plays a primary role in the response to damage induced by acute 4-NQO treatment with PolB2 playing a role in a back-up or ancillary pathway. The lack of sensitivity of the *S. islandicus* LAL14/1 *dpo2* single mutant to either acute or chronic 4-NQO treatment (see below) was surprising in light of a recent report from Feng and colleagues. That study indicated that a *dpo2* knock-out mutant in *S. islandicus* REY15A demonstrated enhanced sensitivity to 4-NQO (Feng et al., 2020). In light of this apparent contradiction, we isolated DNA from the cells plated on the acute 4-NQO treatment plates and confirmed by PCR that the expected genotypes were indeed present (Figures 3D,E). As discussed below, while our data appear at odds with those of Feng and colleagues, they are compatible with a very recent report from Miyabyashi on the phenotypes of DNA polymerase mutants of the congeneric species, *Sulfolobus acidocaldarius* (Miyabayashi et al., 2020).

**FIGURE 4:**
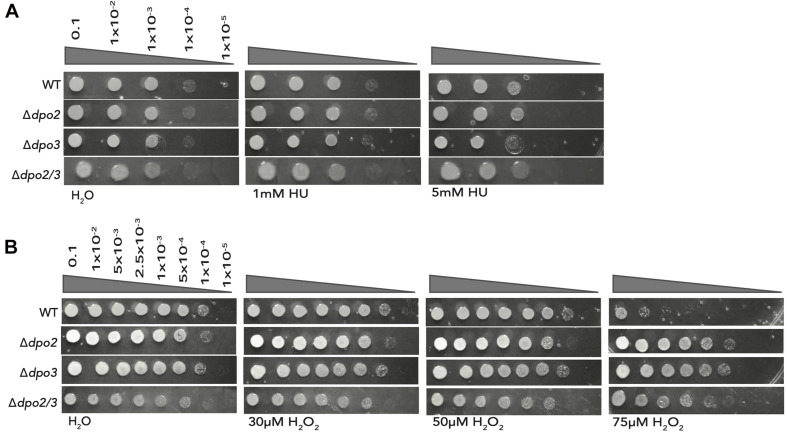
Effect of acute exposure to HU and H_2_O_2_ on strain growth. **(A)** Serial dilutions of each line plated onto TSVYu after growth for 7 h (about 1 doubling time) with HU at the indicated concentrations. Growth conditions and spot plates were repeated at least 3 times. **(B)** Serial dilutions of each line plated onto TSVYu after growth for 7 h (about 1 doubling time) with H_2_O_2_ at the indicated concentrations. Growth conditions and spot plates were repeated at least 3 times.

Next, we tested the effect of acute treatment with hydroxyurea (HU). We have previously demonstrated that this compound induced replication stress and DNA damage and that these effects likely arise as a consequence of the HU-mediated inactivation of the iron-sulfur cluster-containing large subunit of the archaeal primase (Liew et al., 2016). While treatment of the double mutant or the strain lacking PolB3 with 5 mM HU resulted in modestly reduced survival, loss of PolB2 resulted in HU sensitivity indistinguishable from the wild-type cells (Figure 4A).

Finally, we tested the effect of oxidative damage induced by acute treatment with 0, 30, 50, or 75 μM hydrogen peroxide (Figure 4B). Strikingly, loss of either PolB2 or PolB3 caused substantially enhanced survival (~3 orders of magnitude) following treatment with 75 μM H_2_O_2_, The double mutant, in this instance, was less resistant to 75 μM H_2_O_2_ treatment than either single mutant but still displayed 100-fold enhanced survival compared to the wild-type.

### Sensitivity to Chronic DNA Damage

**FIGURE 5:**
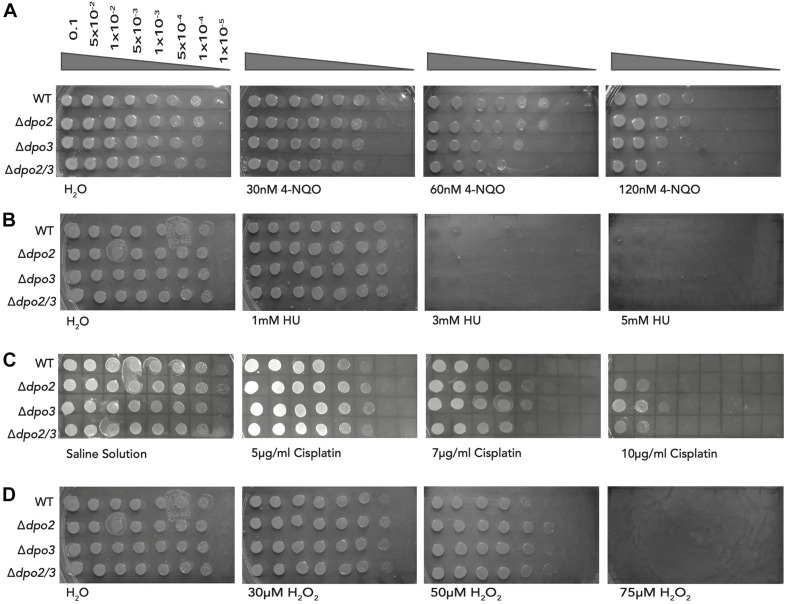
Impact of chronic exposure to 4-NQO, HU, Cisplatin, and H_2_O_2_ on the growth of the strains. **(A)** Serial dilutions of each line plated onto TSVYu containing 4-NQO at the indicated concentrations and grown for 7–8 days. Growth conditions and spot plates were repeated at least 3 times. **(B)** Serial dilutions of each line plated onto TSVYu containing HU at the indicated concentrations and grown for 7–8 days. The positive control plate presented is the same as that seen in Figure 5D (Chronic H_2_O_2_ exposure), both experiments were performed at the same time. Growth conditions and spot plates were repeated at least 3 times. **(C)** Serial dilutions of each line plated onto TSVYu containing Cisplatin at the indicated concentrations and grown for 7–8 days. Growth conditions and spot plates were repeated at least 3 times. **(D)** Serial dilutions of each line plated onto TSVYu containing H_2_O_2_ at the indicated concentrations and grown for 7–8 days. Note that the positive control plate presented is the same as that seen in Figure 5B (Chronic HU exposure), this plate served as positive control for both of the selected replicates of HU and H_2_O_2_ experiments. Growth conditions and spot plates were repeated at least 3 times.

Next, we sought to determine the sensitivity of the wild-type and mutant strains to chronic exposure to the DNA damaging agents tested above. While it was impractical to grow cells under constant exposure to UV light, we tested their ability to grow on plates containing varying concentrations of 4-NQO (Figure 5A). The Δ*dpo2* mutant was very slightly less sensitive to 120 nM 4-NQO than the wild-type cells, the *dpo3* single knock-out was similar to wild-type and the double mutant was more sensitive to 4-NQO than the wild-type.

We additionally tested the effect of chronic exposure to hydroxyurea and could not detect any difference in sensitivity to this agent between wild-type and any of the single or double mutant lines. We note that prolonged incubation of hydroxyurea has been reported to yield a number of toxic breakdown products including cyanide, peroxide and nitric oxide (Kuong and Kuzminov, 2009). Thus, interpretation of these chronic exposure data could be complicated by the multifactorial nature of the stresses imposed upon cells exposed to HU during the 7 days of growth of the organisms on plates.

We also assayed the consequence of chronic exposure to the intra-strand crosslinking agent cisplatin we observed that at low doses of the drug (5 μg/ml) cells lacking either of PolB2 or PolB3 showed modestly enhanced survival when compared to wild-type. This enhanced survival is accentuated at higher cisplatin doses. We note that at 10 μg/ml cisplatin, the single and double mutants show reduced sensitivity when compared to the wild-type strain. We note that we did not report the sensitivity to acute exposure to cisplatin as we found high experimental variability between samples. We were confident that the chronic exposure to cisplatin was yielding equivalent exposures to all cells plated on a given plate.

Finally, we assessed the impact of chronic exposure to hydrogen peroxide. As shown above, acute exposure to this agent yielded a striking increase in survival in cells lacking PolB2 and/or PolB3. Under chronic exposure conditions we observed a modest (maximally 10-fold) enhancement of survival in the mutant lines compared to wild-type at 30 and 50 μM hydrogen peroxide. We note that hydrogen peroxide may have undergone breakdown over the time course of growth on the chronic exposure plates, perhaps consequentially attenuating the phenotype observed.

## Discussion

In agreement with recent reports, we observe that neither PolB2 or PolB3 are required for viability under unperturbed growth condition (Martinez-Alvarez et al., 2017), indeed we observed a modest enhancement of growth rate in both single and double mutant lines. We therefore add to the body of data confirming that PolB1 is the sole B-family DNA polymerase required for genome replication in *Sulfolobus* species. Our data regarding the effect of 4-NQO are largely compatible with the recent report from Miyabyashi, describing a comprehensive analysis of the roles of PolB2, PolB3 and the lesion bypass Y-family DNA polymerase, Dbh, from *Sulfolobus acidocaldarius* (Miyabayashi et al., 2020). However, our data on the role of PolB2 in the response to 4-NQO treatment differ markedly from those of Feng and colleagues, who reported an enhanced sensitivity of the *dpo2* mutants of *Sulfolobus islandicus* REY15A to 4-NQO (Feng et al., 2020). In our experiments in *S. islandicus* LAL14/1, we do not observe any enhanced sensitivity of the *dpo2* mutant strain to either acute or chronic exposure to 4-NQO. In contrast, we actually observe modestly enhanced survival of this mutant line under conditions of chronic exposure to this drug, over a range of concentrations. We note that the predicted amino-acid sequence of PolB2 is identical in the *S. islandicus* strains REY15A and LAL14/1. Significantly, based upon the sequences provided by Feng and colleagues, the Δ*dpo2* strain used in their study differs in the scope of the gene deletion to that of the Δ*dpo2* strain provided to us by Prof. Xu Peng, Copenhagen (Martinez-Alvarez et al., 2017; Feng et al., 2020). The Δ*dpo2* strain utilized in the study by Feng and colleagues does not completely delete the coding region of *dpo2*, leaving the first 21 bp (including the start codon) and final 48 bp (including the stop codon) intact, resulting in a 22 amino acid long peptide “MREMEEY**LRRVYDNVEEVISRC**” the underlined N-terminal seven amino acids correspond to the N-terminal sequences of PolB2 and the 15 residues in bold correspond to PolB2’s C-terminal sequences. It is conceivable that this short peptide could play a trans-dominant negative role (if, for example, the extreme N or C-termini of PolB2 interact with another protein). In contrast, the Δ*dpo2* strain used in our study only has no start codon and only maintains the final 7 bp of the coding region. These differences between the two strains could account for the discrepancy in the data that we observed. The Δ*dpo3* strain used by Feng and colleagues is identical to that provided to us by Prof. Xu Peng. Our deep-sequencing analyses confirm that there are no further mutations in our strains that could reasonably account for this discrepancy in the data. We also emphasize that we re-confirmed the genotype of the strains following the 4-NQO experiment, using DNA obtained from cultures derived from the test plates.

Under conditions of acute treatment with 4-NQO, we observe a 10-fold lower survival of the *dpo3* mutant and loss of both *dpo2* and *dpo3* confers clear 4-NQO sensitivity under both acute and chronic exposure conditions. The data therefore suggest that, under the growth conditions we employed, PolB2 plays a minimal role in recovering from 4-NQO-mediated DNA damage and is secondary to PolB3 in this response.

Arguably the most striking phenotype we observe is the dramatic enhancement of the mutants’ survival upon acute exposure to oxidative damage induced by treatment with hydrogen peroxide. Given that the data discussed above suggest a role for these polymerases in the nucleotide excision repair pathway that repairs damage caused by 4-NQO, it is very surprising that loss of these polymerases stimulates the response to oxidative damage. We note, however, that there is precedent for this sort of phenomenon in the archaeal DNA repair literature. More specifically, Allers and colleagues revealed that loss of the *rad50* and *mre11* genes in *Haloferax volcanii* resulted in enhanced survival compared to the wild-type strain in the presence of a variety of DNA damaging agents, including UV light, ionizing radiation and the alkylating agent methyl methanesulfonate (Delmas et al., 2009). Significantly, it was noted in that study, while overall survival was higher in the *rad50* and *mre11* mutant lines, the kinetics of double strand break repair events were significantly slower. Furthermore, it was proposed that the *Haloferax* RAD50Mre11- containing complex acts to inhibit repair by homologous recombination. We speculate that a conceptually analogous phenomenon of cross-pathway interference may be at play in *Sulfolobus*.

## Data Availability Statement

The datasets presented in this study can be found in online repositories. The names of the repository/repositories and accession number(s) can be found below: NCBI SRA BioProject, Accession No: PRJNA716831.

## Author Contributions

PB performed the experiments. SB and PB designed the experiments and wrote the manuscript. Both authors contributed to the article and approved the submitted version.

## Conflict of Interest

The authors declare that the research was conducted in the absence of any commercial or financial relationships that could be construed as a potential conflict of interest.

## Footnotes

1 https://www.bioinformatics.babraham.ac.uk/projects/seqmonk/
